# Factor structure of the Q-LES-Q short form in an enrolled mental health clinic population

**DOI:** 10.1007/s11136-018-1963-8

**Published:** 2018-09-04

**Authors:** Rachel P. Riendeau, Jennifer L. Sullivan, Mark Meterko, Kelly Stolzmann, Alicia K. Williamson, Christopher J. Miller, Bo Kim, Mark S. Bauer

**Affiliations:** 1 Center for Healthcare Organization and Implementation Research (CHOIR), VA Boston Healthcare System (152M), 150 South Huntington Avenue, Boston, MA 02130 USA; 2 Department of Health Law, Policy and Management, Boston University School of Public Health, 715 Albany Street, Boston, MA 02118 USA; 3 Department of Psychiatry, Harvard Medical School, 2 West, 401 Park Drive, Boston, MA 02215 USA; 4 Department of Anthropology, University of Iowa, 114 Macbride Hall, Iowa City, IA 52242-1422 USA; 5 School of Information, University of Michigan, 4322 North Quad, 105 S. State Street, Ann Arbor, MI 48109-1285 USA

**Keywords:** Quality of life (QOL), Psychometrics, Factor analysis, Q-LES-Q-SF, Mental health, Veterans

## Abstract

**Purpose:**

The Quality of Life, Enjoyment, and Satisfaction Questionnaire-Short Form (Q-LES-Q-SF) is a recovery-oriented, self-report measure with an uncertain underlying factor structure, variously reported in the literature to consist of either one or two domains. We examined the possible factor structures of the English version in an enrolled mental health population who were not necessarily actively engaged in care.

**Methods:**

As part of an implementation trial in the U.S. Department of Veterans Affairs mental health clinics, we administered the Q-LES-Q-SF and Veterans RAND 12-Item Health Survey (VR-12) over the phone to 576 patients across nine medical centers. We used a split-sample approach and conducted an exploratory factor analysis (EFA) and multi-trait analysis (MTA). Comparison with VR-12 assessed construct validity.

**Results:**

Based on 568 surveys after excluding the work satisfaction item due to high unemployment rate, the EFA indicated a unidimensional structure. The MTA showed a single factor: ten items loaded on one strong psychosocial factor (*α* = 0.87). Only three items loaded on a physical factor (*α* = 0.63). Item discriminant validity was strong at 92.3%. Correlations with the VR-12 were consistent with the existence of two factors.

**Conclusions:**

The English version of the Q-LES-Q-SF is a valid, reliable self-report instrument for assessing quality of life. Its factor structure can be best described as one strong psychosocial factor. Differences in underlying factor structure across studies may be due to limitations in using EFA on Likert scales, language, culture, locus of participant recruitment, disease burden, and mode of administration.

## Introduction

Clinical, research, and administrative interest in quality of life (QOL) has increased over the past three decades. QOL is generally defined as an individual’s subjective, holistic view of life circumstances across physical, psychological, and social domains [1–4]. It is a valuable predictor of patients’ overall health status, their perceptions of health services, and how to improve those services [3, 5, 6]. To that end, measures of patient-reported outcomes that emphasize patients’ subjective perspectives like QOL are increasingly used in psychiatry [6] and relevant given the growing attention to self-defined recovery as the goal of healthcare [7]. Many tools that measure QOL have been developed and examined to determine their reliability, validity, feasibility, and sensitivity to change [8].

One such self-report tool is the Quality of Life Enjoyment and Satisfaction Questionnaire (Q-LES-Q), designed to measure a patient’s satisfaction and enjoyment in different areas of daily functioning. The original scale consists of 93 questions, which were grouped into eight subscales on the basis of expert clinical opinion: physical health, subjective feelings, leisure time activities, social relationships, work, school/coursework, household duties, and general activities [9]. The abbreviated version (Q-LES-Q-SF) consists of 14 items derived from the long form’s general activities subscale, plus two questions about medication and overall life satisfaction. Both versions are among the most frequently used QOL measures in psychopharmacology and clinical trials [10], and have been translated into several languages.

A number of studies have assessed the reliability, validity, and factor structure of the Q-LES-Q and Q-LES-Q-SF to date (see Table 1). Notably, there is little consensus regarding the factor structure for the Q-LES-Q-SF [10], with research indicating one factor [2, 9], two factors [5], or an equal chance of one or two factors [11]. Psychometric studies have involved work with versions in Chinese, French, and other languages and cultures, which may explain the lack of consensus in the results [2, 3]. A variety of methods (exploratory factor analysis using Pearson’s or polychoric correlations, principal components analysis, confirmatory factor analysis, structural equation modeling) may have contributed to diverse findings [2, 5, 9, 11]. Moreover, investigations of the psychometric properties of the Q-LES-Q-SF in English have been sparse, and none have addressed its factor structure.

**Table 1:** Review of peer-reviewed literature on the Q-LES-Q (all forms)

Study^a^	Language and country	Location, diagnosis, mode of administration^b^	*n*	Mean age (years)	% Female	Overall alpha	Factor structure	Test–retest reliability		Convergent validity (with total score unless otherwise noted)
Short form: Q-LES-Q-SF										
Rapaport [1]	English US	C, O, O (mail)	67	32.4	65.8%	0.90	–	At 1–2 weeks (ICC = 0.86)^c^		–
Mick [2]	English US	Mx, Mx, I	150 ADHD^d^	36.2 ± 10.8	46% (*n* = 81)	0.88	–	–		SAS total T (*r* = 0.72)
			134 Non-ADHD adults	30.0 ± 8.7	53% (= 63)	0.84		–		CGI
			173 Placebo	37.1 ± 9.5	47% (*n* = 91)					CGI
Wyrwich [3]	English US	O, P, I	2588	40.0 ± 12.1 (Range 18–65)	64.6%	0.86	–	–		HAM-A, CGI-S, MADRS, PSQI: Week 1(*r* = − 0.25 to − 0.36), Week 8 (*r* = − 0.25 to − 0.36)
Stevanovic [4]	Serbian Serbia	I, P, I	57	47.16 ± 9.22	33.3%	0.90	All items but work (3) correlated to total (*r* = 0.41–0.81)	At 1 week (ICC = 0.93, *n* = 54)		CGI-S (*r* = 0.89) PGIs (*r* = 0.43) CGI-I (*r* = 0.47)
Lee [5]	Chinese Taiwan	O, M, I	1482	54.65	58.57%	0.87	EFA, CFA^e^ 2 factors Psychosocial (eigenvalue = 5.24, 37.41% variance) Physical (eigenvalue = 1.27, 9.07% variance)	At 2 weeks (ICC = 0.75, *n* = 199)		SF-12 (*r* = 0.35–0.38) PHQ-9 (*r* = 0.37) HAM-D-17(*r* = 0.49) *Psychosoc. subscale*: MCS-12 (*r* = 0.37) PCS-12 (*r* = 0.27) *Physical Subscale*: MCS-12 (*r* = 0.28)

Study	Language and country	Location, diagnosis, mode of administration	*n*	Mean age (years)	% Female	Overall alpha	Factor structure	Test–retest reliability	Convergent validity (with total score unless otherwise noted)
McEvoy [6]	English Australia	O, Mx, I	843	32.06	78.4%	0.88	–	–	–
Ishak [7, 8]	English US	E, P, I	4041	42.6 ± 13	62.8%	0.87	–	(ICC = 0.74)	–
Bourion-Bédès [9, 10]	French France	O, S, I	124	39.2 ± 11.2 Median 36	16.7%	0.90	CFA 1 factor: (RMSEA = 0.077) (90%CI [0.054–0.098]),CFI = 0.968 TLI = 0.962, loadings (*r* = 0.523 and 0.851) CFA 2 factors: (RMSEA = 0.076) (90%CI [0.054–0.098]), CFI = 0.969 TLI = 0.963, loadings (*r* = 0.525 and 0.870) (a) PSI high for unidimensional construct (IRT = 0.902, item residuals − 2.5 to + 2.5) All items significantly correlated to total (*r* = 0.47–0.79) (b)		
Long-form Q-LES-Q									
Endicott [11]	English US	O, P, I	95	39.1 ± 10.7 Range 18–63	59%	–	General activities (GA)^f^ (*α* = 0.90, *n* = 83) Other subscales (*α* = 0.92–0.96)	GA (*r* = 0.74, *n* = 54) Other subscales (*r* = 0.63–0.89)	HAM-D: (*r* = − 0.61), SCL-90 (*r* = − 0.67) BDI^b^ (*r* = − 0.36) *Subscale level* CGI-S: (*r* = 0.34–0.68) GA (*r* = − 0.66)
Bishop [12]	English US	I, P, I	151	18–65	55%	0.6	PCA^g^ on 44 items from 4 original domains confirmed 4 subscales Physical health (*α* = 0.93), Subjective feelings (*α* = 0.95,) Leisure activities (*α* = 0.89), Social relationships (*α* = 0.89)		
Rossi [13]	Italian Italy	O, P, I	404	38.9 ± 12.3	55.9%	–	GA (*α* = 0.92) Other subscales (*α* = 0.82–0.96)	At 1 week (ICC > 0.8, *n* = 62) GA (ICC = 0.89, *n* = 62)	WSAS (*r* = − 0.56)
Schechter [14]	English US	C, O (healthy), I	529	33 Range 18–84	58%	–	GA (*α* = 0.90, *n* = 69); correlated with other subscales (*r* = 0.41–0.62); Other (*α* = 0.82–0.93)	GA (ICC = 0.86, *n* = 69) Other subscales (ICC = 0.58–0.89, *n* = 69)	–

Study	Language and country	Location, diagnosis, mode of administration	*n*	Mean age (years)	% Female	Overall alpha	Factor structure	Test–retest reliability	Convergent validity (with total score unless otherwise noted)
Zubaran [15]	Portuguese Brazil	I, S, I	100	20.1 ± 6.2	22%	–	GA (*α* = 0.84) Other subscales (*α* = 0.78–0.93)	*WHOQOL-BREF areas with subscales* Physical: physical (*r* = 0.46), feelings (*r* = 0.54), leisure (*r* = 0.28), GA (*r* = 0.6) Psychological: physical (*r* = 0.4), feelings (*r* = 0.6), leisure(*r* = 0.34), social (*r* = 0.49), GA(*r* = 0.61) Social: physical (*r* = 0.3), feelings (*r* = 0.48), social (*r* = 0.44), GA (*r* = 0.48) Environment: physical (*r* = 0.37), feelings (*r* = 0.5), household (*r* = 0.3), social (*r* = 0.4), GA (*r* = 0.5)	
Pitkänen 2012 [16]	Finnish Finland	I, P, I	190	38 ± 13 (range 18–65)	41%	0.89	One factor (41% variance) Three factors (56% variance)	EQ-5D (*r* = 0.445; *p* < 0.001)	
Other forms									
Ritsner [17–19]	Hebrew Israel	Mx, P, I	339 Initial 133 Confirm. 175 control 133 Model construction 124 follow-up 38 Inpatient	38.5 ± 10.3 39.6 ± 9.3 38.4 ± 9.9 39.6 ± 9.3 39.9 ± 9.4 36.6 ± 8.6	29.2% 23.3% 72.9% 23.3% 21.8% 18.4%	0.92 (GA)	18-Item scale of Long-Form EFA 4 factors (*α* = 0.74–0.97) Q-LES-Q domains (*α* = 0.69–0.87)	At 2 weeks: (ICC = 0.79–0.90)	QLS (*r* = 0.40–0.65, *p* < 0.001) (b)

^a^See references for full citations

^b^Location: *I* inpatient,* O* outpatient,* C* community sample,* E* enrolled population,* Mx* mixed,* O* other,* Unk* unknown. Diagnosis:* P* psychiatric,* M* medical or primary care,* S* substance abuse,* Mx* mixed,* O* other,* Unk* unknown. Mode of administration:*I* in person, *T* telephone, *W* web, *O* other, *Unk* unknown, *BDI* Beck Depression Index

^c^ICCC stands for inter-class correlation

^d^Scales abbreviation key in order of appearance: Attention Deficit Hyperactivity Disorder, Social Adjustment Scale, Clinical Global Impression Scale, Hamilton Anxiety Rating Scale, Clinical Global Impression-Severity scale, Montgomery–Åsberg Depression Scale, Pittsburgh Sleep Quality Assessment, Patient Global Impression of Severity, Patient Health Questionnaire, Hamilton Rating Scale for Depression, Short Form-12 (SF-12): Mental Component Score, Physical Component Score, Symptom Checklist-90, Beck Depression Index, Work and Social Adjustment Scale, World Health Organization Quality of Life Brief form, Quality of Life Scale (Hebrew)

^e^Confirmatory factor analysis, exploratory factor analysis, Short Form-12 (SF-12): Mental Component Score, Physical Component Score, Confirmatory Factor Analysis/Index, Root Mean Square Error of Approximation, Confidence Interval, Tucker–Lewis Index, Person Separation Index, Item Residual T

^f^General activities (GA) is a subscale of the long-form Q-LES-Q with the same questions as the Q-LES-Q-SF

^g^Principal components analysis

In addition, samples used for the majority of Q-LES-Q-SF factor analyses have been recruited during an episode of inpatient or outpatient care or when patients arrived at medical facilities for treatment. It is unclear whether the factor structure remains stable for populations who complete the survey removed from the point of care, that is, outside of a clinic and not necessarily actively care-seeking. Testing the factor structure on patients outside the point of care would thus entail analysis in a more diverse sample of patients. Measuring QOL within this population is important for population-based healthcare systems, such as national health services and accountable care organizations (ACOs) which must proactively manage patient care, as well as for payers and organizations monitoring healthcare delivery [11, 12]. Furthermore, there are no psychometric data on telephone administration of this survey which allows for interactive self-reports outside the point of care. Given the variation in Q-LES-Q-SF results to date and the limitations in the populations studied, the purpose of our current study is to advance understanding of the possible reasons for previous findings of both uni- and bi-dimensional latent structures elsewhere by exploring possible Q-LES-Q-SF factor structures in a population of individuals outside the point of care enrolled in treatment. General mental health clinics across nine United States Department of Veteran Affairs medical centers (VAMCs) provide treatment. These analyses aim to contribute to understanding the dimensionality of the Q-LES-Q-SF in enrolled populations with varied psychiatric diagnoses and physical comorbidities and to the sparse literature on the reliability of the English language version.

## Methods

This study was approved by the VA Central Institutional Review Board. We obtained a waiver of written informed consent and obtained verbal informed consent from all individual participants included in the study. Data were collected at baseline for a controlled implementation trial that focused on evidence-based team care and its downstream impact on healthcare outcomes and satisfaction [13].

### Sample and data collection

The study population consisted of Veterans who had at least two behavioral health visits in the prior year (with at least one visit within the past three months) to a mental health clinic at one of the nine VAMCs, excluding those who received a diagnosis of dementia during this interval (*n* = 5596 as the sample frame). From these, up to 500 individuals from each VAMC were randomly selected for telephone interviews, up to 85 per site, with a total goal of 765 participants at baseline based on power calculations for the original trial. Women were oversampled for gender balance. Potential participants received opt-out instructions if they chose not to be called and study information in the mail 2–6 weeks prior to calls. Non-clinician phone interviewers received extensive training including assigned readings, role-playing, supervised full-length practice interviews, frequent peer conferencing, and access to a study clinician in order to standardize administration. Interviewers administered the survey battery to Veterans over the telephone in English. Potential participants who could not be reached after three calls were excluded, resulting in a total sample of 576 completed interviews.

### Instruments

The Q-LES-Q-SF’s 16 self-report items evaluate overall enjoyment and satisfaction with physical health, mood, work, household and leisure activities, social and family relationships, daily functioning, sexual desire/interest/performance, economic status, vision, ability to get around physically, overall well-being, medications, and contentment [13]. Items are rated on a five-point Likert scale (“very poor,” “poor,” “fair,” “good,” “very good”), with higher scores indicating better enjoyment and satisfaction with life. The scoring of the Q-LES-Q-SF involves summing the first 14 items to yield a total score. The last two items about medication and overall contentment were added to the short form for clinical reasons and are scored separately [10]. The total score ranges from 14 to 70 and is expressed as a percentage based on the maximum total score of the items completed (0–100). The normal range that represents community sample scores is 70–100 [6, 9].

To measure construct validity of the Q-LES-Q-SF, we compared it to a similar QOL assessment with clearly established factor structure. The Veterans RAND 12-Item Health Survey (VR-12) was adapted for Veteran populations from the SF-12, an abbreviated version of the SF-36 [14–16]. This 12-item health status measure contains two summary ratings: the Mental Component Score (MCS) and Physical Component Score (PCS). The MCS and PCS provide an assessment of overall mental and physical health status, respectively, over the past month. Each score ranges from 1 to 100, with a higher score indicating a more favorable health status.

We also asked background questions regarding current employment status and race/ethnicity. As part of the interview protocol, we administered a harm risk screener consisting of the Patient Health Questionnaire (PHQ)-9 and, if appropriate, the P4 Screener, both used for suicide risk stratification [17]. Additional demographic and diagnoses data such as service utilization were gathered from the VA Corporate Data Warehouse (CDW).

### Psychometric analyses

The analysis proceeded by testing the reliability and validity of the Q-LES-Q-SF items using a random split-sample (n=288 and n=288), a common psychometric approach [18, 19], to conduct an exploratory factor analysis (EFA) followed by multi-trait scaling analysis (MTA) to validate the EFA, as we have done in prior work [20]. We started with EFA, given the conflicting findings regarding the dimensional structure of the instrument (i.e., both single- and dual-factor solutions) [21, 22]. We used oblique promax rotation (i.e., allowing the factors to correlate). The EFA calculates communalities, which represent the level of shared variance of each item with the other items. Communalities are considered low if *r* < 0.4, moderate if between 0.4 and 0.79 inclusive, and high if *r* ≥ 0.8 [23].

Two rules were applied to decide the number of factors to retain and rotate: the Kaiser–Guttman rule (i.e., factors with eigenvalues > 1), and Cattell’s scree plot method [20]. While these criteria used along are not recognized as best practice for deciding the number of factors to retain [24–26], they have nonetheless been widely applied, and their use here allowed us to explore both single- and dual-factor solutions to compare with the literature. To supplement these approaches, however, we also applied two additional methods recognized as best practice [24] to further inform the number of factors to retain and rotate: the full sample (n = 568): Velicer’s minimal average partial (MAP) and Horn’s parallel analysis (PA) using simulation [27].

To explore the EFA-derived structure in view of existing two-factor solutions [2, 6], we ran an MTA on the other half sample [28, 29]. MTA, based on Campbell and Fiske’s [30] multi-trait, multi-method approach, assesses the pattern of correlations between all questionnaire items and the hypothesized scale scores computed from those items [31, 32]. Validity of the theorized scales is determined through the pattern of convergence and discrimination among the correlations. MTA begins with the assignment of each item to a hypothesized scale; items with EFA loadings ≥ 0.40 on two different factors were provisionally assigned to the factor with the highest loading. Item convergent validity was considered adequate if there was at least a 0.30 correlation between the item and its hypothesized scale. Item discriminant validity (i.e., scaling success) was judged to be supported if the correlation between an item and its hypothesized scale was higher (probable success) or significantly higher (definite success) than the correlation between that item and any other scale. Item discriminant validity was not supported (i.e., scaling failure) if the correlation between the items was more strongly correlated with other scales rather than its hypothesized scale, representing probable or definite scaling failures, respectively. Significance testing was two-tailed; we used the 0.05 level of tolerance for Type 1 error as our criterion for statistical significance [33].

Based on the empirical evidence, MTA results for the initial hypothesized scale structure and conceptual considerations, items were reassigned to other factors or dropped from the analysis to improve convergent validity (consistency within subscales) and discriminant validity (distinctions between factors). We then ran the MTA again using the revised item-to-scale assignments, and the cycle was repeated until no further improvements in validity were achieved. Cronbach’s alpha coefficients were computed as part of MTA and monitored throughout the process of item reassignment to ensure adequate scale internal consistency reliability for group comparisons (*α* ≥ 0.70) [20]. Floor and ceiling effects were measured and considered acceptable if fewer than 15% of respondents answered with the lowest or uppermost answer option [34].The internal consistency of the final overall Q-LES-Q-SF and resultant subscales were assessed using the Cronbach’s alpha coefficient. Internal consistency was classified as satisfactory at 0.70–0.79, good from 0.80 to 0.89, and excellent at ≥ 0.90 [16].

To determine if clustering at the medical center level occurred among scores beyond slight variation due to expected regional differences [17], we calculated inter-class correlations (ICC). ICC(1) provides an estimate of the reliability of one respondent’s score as an estimate of a relevant group mean; values between 0.05 and 0.30 are typical indicators of clustering [34].

Construct validity of the overall instrument and its subscales was assessed by correlations between the Q-LES-Q-SF factors and the VR-12 MCS and PCS using Pearson’s correlation coefficients. All statistical analysis was conducted using SAS software, Version 9.4 [35].

## Results

### Demographics

Eight respondents of the 576 Veterans surveyed did not answer any Q-LES-Q-SF items and were removed. Of the remaining 568 Veterans, 20.4% were female, with a mean age of 54.2 ± 13.7 years (Table 2). Of the sample, 18.2% worked full-time, 4.6% worked part-time, 6.7% were unemployed, 35.1% were retired, and 30.0% were disabled. The most common mental health diagnoses were post-traumatic stress disorder (PTSD) (48.2%) and major depressive disorder (46.1%). The most common medical diagnoses were pain (49.5%) and hypertension (35.1%). The average score on the full Q-LES-Q-SF scale was 53.28 ± 19.4 (Table 3). Minimal floor and ceiling effects were observed (< 15%).

**Table 2:** Population demographics (*n* = 576)

Demographic characteristics	*n* (%)
Age (years)	568
Mean ± standard deviation	54.2 ± 13.71
Range	22.98–94.49
Gender	
Female	116 (20.4)
Male	454 (79.6)
Race	
White	438(81.6)
Black	86 (16.0)
Other	13 (2.4)
Ethnicity	
Hispanic or Latino	49 (8.9)
Not Hispanic or Latino	499(91.1)
Service connected disability	
≥ 50%	347 (78.9)
Employment status	
Full-time	103 (18.2)
Part-time (> 30 h)	26 (4.6)
Leave of absence	2 (0.4)
Unemployed (economic)	38 (6.7)
Disabled	199 (35.1)
Retired	170 (30.0)
Student (full or part-time)	5 (0.9)
Homemaker	21 (3.7)
Other	2 (0.2)
Marital status	
Married	267 (47.3)
Never married	78 (13.8)
Divorced, Widowed, or separated	219 (38.8)
Suicidality Risk (PHQ9, [19])	
No risk	229 (80.9)
Minimal risk	8 (2.8)
Lower risk	33 (11.6)
Higher risk	14 (4.9)
Mental health diagnoses	
Anxiety disorder	189 (33.3)
Major depressive disorder	262 (46.1)
PTSD	274 (48.2)
Schizophrenia	28 (4.9)
Bipolar disorder	173 (40.5)
Personality disorder	31 (5.5)
Substance abuse disorder	97 (17.1)
Medical diagnoses	
Pain	281 (49.5)
AIDS	3 (0.5)
Cancer	18 (3.2)
Stroke	4 (0.7)
Lung	70 (12.3)
Diabetes	125 (22.0)
Liver	15 (2.6)
Hypertension	201 (35.1)
Myocardial infarction	49 (8.9)
Obesity	98 (17.3)
TBI	8 (1.4)
Tobacco use	72 (12.7)
Comorbid medical diagnoses	
Any	96.65
Two	17.96
Three or more	66.2
Service utilization	
Medical/surgical inpatient admission in prior year	24 (4.2)
Medical/surgical inpatient admission in prior year	39 (6.9)

**Table 3:** Descriptive statistics and exploratory factor analysis (EFA) loadings for the Q-LES-Q-SF in an enrolled mental health clinic U.S. Veteran population (*n* = 568)

Item: In the past two weeks, how satisfied have you been with…	Mean ± SD (n = 568)	Single-factor EFA loadings (n = 234)	Dual-factor EFA loadings (n = 234)	
*Entire scale*	53.28 ± 19.38	*–*	*Factor 1*	*Factor 2*
1. …physical health?	2.92 ± 1.18	0.70*	0.43*	0.39
2. …mood?	2.90 ± 1.17	0.73*	0.72*	0.05
3. …work?	3.16 ± 1.28	N/A		
4. …household activities?	2.91 ± 1.22	0.74*	0.66*	0.14
5. …social relationships?	2.82 ± 1.35	0.73*	0.75*	0.01
6. …family relationships?	3.44 ± 1.25	0.56*	0.65*	-0.08
7. …leisure time activities?	2.99 ± 1.26	0.71*	0.70*	0.04
8. …ability to function in daily life?	3.22 ± 1.10	0.75*	0.56*	0.29
9. …sexual desire, interest, and/or performance?	2.43 ± 1.33	0.42*	0.18	0.33
10. …economic status?	3.05 ± 1.20	0.48*	0.50*	− 0.02
11. …living/housing situation?	3.75 ± 1.12	0.49*	0.40*	0.13
12. …ability to get around physically without being dizzy or unsteady or falling?	3.49 ± 1.25	0.58*	− 0.13	1.02*
13. …your vision in terms of ability to do work or hobbies?	3.54 ± 1.16	0.38	0.08	0.42*
14. …overall sense of well-being?	3.25 ± 1.06	0.80*	0.65*	0.22
*Eigenvalues*	N/A	10.55	10.56	1.07

Full-scale range of responses was 1.92–100. Individual item range was 1.00–5.00. Item 3 excluded due to low response rate. Item discriminant validity was 92.3% for dual-factor solution

*Correlations greater than 0.4

We excluded Item 3 on the Q-LES-Q-SF (work) from analyses due to very low employment rates (71.8% unemployed, disabled, or retired; Table 2). Respondents with greater than 50% of items missing were removed from each half sample, resulting in (n = 234) for EFA and (n = 283) for MTA.

### Explanatory factor analysis

The EFA using oblique promax rotation yielded one or two factors (Table 3). Eigenvalues were strong for Factor 1 (10.56) and borderline for Factor 2 (1.07) in the dual-factor solution, which we pursued further for comparison with prior literature. The scree plot mirrored this finding. Ten items loaded on Factor 1 and two items loaded on Factor 2. Although item 9 (sex) loaded on Factor 2 more strongly than on Factor 1, it did not meet the 0.40 cut-off for either factor (Table 3). Communalities were low for items 6, 9, 10, 11, and 13 (*r* < 0.4) and markedly high for item 12 (ability to get around physically: *r* = 0.91). However, the Minimal Average Partial and Horn’s parallel analysis suggested a single factor.

### Multi-trait analysis

In view of the borderline EFA results and prior literature [2, 6, 16, 36], the MTA was used to explore goodness of fit of the 2-factor model. The internal consistency of the entire Q-LES-Q-SF was substantial and consistent with prior work (*α* = 0.85). Initially, based on conceptual considerations, and a desire to use as many of the available items as possible, we placed item 9 (sexual desire) with items 12 (ability to get around physically) and 13 (vision) together to constitute a “physical” subscale despite item 9’s overall lack of substantial loading in the EFA. The other factor, a “psychosocial” subscale, included the 10 items that loaded on Factor 1. Item 1 (physical health) was also moved to the “physical” subscale based on face validity. Internal consistency of the psychosocial and physical subscales was strong to moderate (*α* = 0.85 and 0.68, respectively). No substantial increase in alphas for either scale was achieved by eliminating any item. Despite these promising results, the initial MTA yielded low significant item discriminant validity (46%), in large measure due to item 9 in the proposed physical subscale. Therefore, we reassigned item 9 to the psychosocial factor.

Our final MTA (Table 4) incorporated item 9 (sex) into the psychosocial subscale, and placed items 1 (physical health), 12 (ability to get around physically), and 13 (vision) into the physical subscale. This eliminated item-level discriminant validity failures for all items and raised the scale’s significant item discriminant validity from 46% to 92%. Internal consistency was strong for the psychosocial subscale (*α* = 0.87), and just below satisfactory for the physical subscale (α = 0.63). Alpha-if-item-deleted statistics from the second MTA indicated that no substantial gains in internal consistency could be achieved by eliminating any item. The inter-scale correlation improved (i.e., was considerably lower) compared to that observed in the initial MTA (*r* = 0.50 versus initial *r* = 0.60). Item discriminant validity was strong at 92.3%.

**Table 4:**
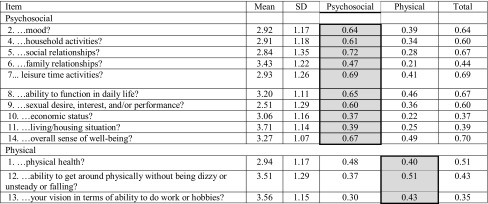
Multi-trait scaling analysis (MTA): item descriptive statistics and item-to-scale correlations

Shading indicates factor assignment of each item. MTA run on split sample (*n* = 283)

Across all nine sites, means of responses were 51.96 ± SD20.18 for the psychosocial factor and 58.45 ± SD22.88 for the physical factor. The inter-class correlations ICC (1) did not show clustering for the full scale (0.02), the two subscales (psychosocial = 0.01 and physical = 0.03), or for any item (− 0.003–0.047).

### Construct validity

We evaluated construct validity by comparing VR-12 scores with the full sample of Q-LES-Q-SF survey respondents (*n* = 568). The mean VR-12 PCS score was 35.33 ± 13.08 and the mean MCS score was 36.69 ± 14.72. The full Q-LES-Q-SF demonstrated moderate correlation with the MCS (*r* = 0.65; *p* < 0.001) and weaker correlation with the VR-12 PCS (*r* = 0.38; *p* < 0.001). Notably, the PCS showed a significant moderate correlation with the physical subscale we propose (*r* = 0.54; *p* < 0.001), compared to a weaker correlation with the psychosocial subscale (*r* = 0.28; *p* < 0.001). Conversely, the MCS correlated with moderate to strong significance with the psychosocial scale (*r* = 0.68; *p* < 0.001) compared to the physical scale (*r* = 0.35; *p* < 0.001).

## Discussion

We investigated the factor structure of the Q-LES-Q-SF based on data collected during telephone interviews with 568 Veterans enrolled in general mental health services but not at the point of care at the time of survey completion. Notably, our sample had substantial physical and mental health burden (Table 2). Using EFA and MTA in a split-half approach to consider both uni- and bi-dimensional solutions, we identified single- and dual-factor solutions, the latter with a strong psychosocial factor (*k* = 10) and a possible weaker physical health factor (*k* = 3).

Interpreting our bi-dimensional results is informed by reviewing the two prior studies of Q-LES-Q-SF factor structure, one in Chinese [6] and the other in French [2, 16]. The former, using a primary care locus of recruitment, identified both a psychosocial and a physical factor, while the latter, recruiting from a substance abuse facility, identified a single, overall factor and a possible second factor. Ethnic/cultural variability in the expression of general mental illnesses, particularly major depressive disorder, is well documented; for example, compared to depressed Western populations, Chinese patients often endorse physical rather than psychological symptoms [37]. Such tendencies may partly explain the difference in the Q-LES-Q-SF factor structure identified by Chinese and French studies. Further, the degree to which differences in language, culture, locus of sample recruitment, or a combination of these factors contribute to the differences in findings between those previous studies and the present one cannot be determined for certain.

Our results provide insight into this divergence of findings. Similar to both prior studies, we found a strong primary factor in our sample representing a psychosocial subscale. The physical health factor was much weaker and, in our estimation, equivocal. Recalling that the original Q-LES-Q-SF was constructed based on expert opinion without formal psychometrics, it is not surprising to observe some instability in factor structure across languages and populations. However, from our and prior [2, 6] analyses, it is clear that a strong psychosocial factor can be distinguished. In fact, relying solely on the EFA results, one could make a strong case for a unidimensional factor—Factor 1 in this study—which also includes item 1 (physical health). Indeed, the MAP and Horn’s Parallel Analysis tests support the existence of a single factor.

In contrast, the presence of a separate, distinctly physical health factor is uncertain. The Q-LES-Q was initially reported in the psychopharmacologic literature [9] and extensively used in medication treatment trials. The three specific physical health items included by experts (physical ability, vision, sex) correspond to side effects frequently encountered in the medications typically investigated at that time (tricyclic antidepressants and first generation antipsychotics), and this may be a reason behind the inclusion of these specific symptoms. Changes in medication usage and their side effects in the years since the introduction of the Q-LES-Q, combined with differences in population characteristics across studies, may mitigate the usefulness of these three items to distinguish differences in patient experience.

Nonetheless, the pattern of convergent and discriminant validity of the proposed Q-LES-Q-SF factors with the VR-12 suggest the possibility of a two-factor structure. The proposed Q-LES-Q-SF psychosocial factor correlates more strongly with the MCS than the PCS. Conversely, the proposed Q-LES-Q-SF physical factor correlates more strongly with the PCS than the MCS.

Correlations of the full Q-LES-Q-SF with the MCS and PCS reveal differences in relative strength that suggest a unidimensional interpretation that emphasizes the psychosocial content of the measure. The Q-LES-Q-SF overall score correlated strongly with the VR-12 MCS and less so with the PCS. Consistent with our findings, Bourion-Bédès and colleagues’ [2] also found a strong correlation between Q-LES-Q-SF total score and the MCS from the SF-12 (from which the VR-12 derives), and weaker correlation with the SF-12 PCS. In Lee and colleagues’ [6] study, their psychosocial factor correlates more strongly with the MCS, and the physical factor with the PCS as in Bourion-Bédès’ and our Western samples. The entire Q-LES-Q-SF in Lee’s study shows modest, nearly identical correlations with both the SF-12 PCS (*r* = 0.35) and MCS (*r* = 0.38).

All three studies, across three distinct populations, cultures, and languages, converge around a strong psychosocial factor. In the present study and that of Lee and colleagues [6], this is complemented by a weaker, separate physical factor, while Bourion-Bédès and colleagues’ study [2, 16] resulted in a single overall factor that is heavily psychosocially weighted.

Thus researchers and program evaluators can have confidence in using the Q-LES-Q-SF as a single, psychosocial factor. The two-factor solution can be used with little confidence due to equivocal support for a distinct physical factor in the measure as it currently exists.

## Limitations

Our study has several limitations. We used Pearson’s rather than polychoric correlations to allow for closer comparison to Lee and colleagues (2014) bi-dimensional results. Future research in this area could focus on alternative methods utilizing polychoric correlations for ordinal scales. We also note that the Kaiser criterion and scree plot method used here to mirror the procedures used by Lee and colleagues (2014) and many others may lead to overdimensionalization, especially when Likert scales are involved [24–26]. To at least partially mitigate this possibility, we also applied Velicer’s minimal average partial (MAP) and Horn’s parallel analysis (PA) and recommend similar multiple procedures be used in future research.

We also had high missing data rate for item 3 (work), likely due to high rates of retirement, disability, and unemployment in our sample. However, this has been found in other studies involving mental health and substance use populations [2, 4, 10, 16, 38]. Following Bishop and colleagues’ [5] example, we excluded questions related to work because morbidity rates in their populations were too high.

It is also possible that mode of administration affected results. Patients may be less willing to disclose about sensitive topics in real-time conversation compared to mail-out surveys. However, both Lee and colleagues [6] and Bourion-Bédès and colleagues [2, 12] administered surveys within clinics, which may feel even less anonymous than phone interviews. Additionally, paper administration for clinical purposes includes instructions on circling specific facets of the topic in question that cause dissatisfaction within items, i.e., Item 9: “sexual desire, interest, and/or performance” (Endicott, personal communication). When the survey is administered aloud, all three elements of sexual experience must be mentally combined in some fashion and judged together. Similar conflicting interpretations of questions with options may explain the low communalities of items 11 (“living *or* housing situation?”) and 13 (“vision, in terms of work *or* hobbies?”) and the unusually high communality for item 12 (“able to get around without feeling dizzy *or* unsteady *or* falling?”). Although the aspects of items chosen on paper do not affect the total Q-LES-Q-SF score, the options inherent in the items mean that these responses may be more variable than other items.

The higher proportion of males within our sample may have also affected how item 9 (sex) loaded in factor analyses. Even when oversampling for females, 79.6% of the participants were male (Table 1). For item 9 in particular, differential item functioning has been observed based on sex [2].

Finally, our study may be limited in that results may differ in populations treated in systems different from the VA, a large, publicly funded healthcare system. Patients receiving care within the VA comprise an aging population with a high proportion of males who have high rates of physical comorbidities [39]. These comorbidities are shown to contribute to perceptions of QOL and overall health outcomes [40]. However, this type of population will become increasingly relevant as a higher proportion of the world-wide population ages and as general mental health care becomes more integrated into primary and specialty services [41–43].

## Conclusions

This study confirms that the Q-LES-Q-SF is a valid and reliable recovery-oriented self-report instrument within a general mental health population assessed not at the point of care. The factor structure may be best described as one clear psychosocial factor and one possible weaker physical factor, a structure which may vary according to factor structure extraction methods, treatment of Likert scales as ordinal versus categorical, degree of disease burden, culture, language, and mode of administration. While the psychosocial factor is notably stable across three populations, cultures, and languages, future research may reduce the instability of the second physical factor, perhaps even incorporating additional items. In addition, further assessment of the effect of administration mode (i.e., paper versus phone) on the Q-LES-Q-SF responses and factor structure is needed.

Based on current evidence, researchers and program evaluators can be secure in using the full Q-LES-Q-SF score or the single 10-item psychosocial factor. In the evaluation of interventions or other studies with a particular focus on the physical quality of life, breakout scoring of the physical subscale might be examined with considerable caution. However, in those circumstances where the assessment of physical quality of life is critical, the use of supplemental validated measures of that dimension is recommended.
